# SARS-CoV-2 neutralizing antibody responses are more robust in patients with severe disease

**DOI:** 10.1080/22221751.2020.1823890

**Published:** 2020-09-25

**Authors:** Pengfei Wang, Lihong Liu, Manoj S. Nair, Michael T. Yin, Yang Luo, Qian Wang, Ting Yuan, Kanako Mori, Axel Guzman Solis, Masahiro Yamashita, Ankur Garg, Lawrence J. Purpura, Justin C. Laracy, Jian Yu, Leemor Joshua-Tor, Joseph Sodroski, Yaoxing Huang, David D. Ho

**Affiliations:** a Aaron Diamond AIDS Research Center, Columbia University Vagelos College of Physicians and Surgeons, New York, NY, USA; b Division of Infectious Diseases, Department of Internal Medicine, Columbia University Vagelos College of Physicians and Surgeons, New York, NY, USA; c Department of Cancer Immunology and Virology, Dana-Farber Cancer Institute, Department of Microbiology, Harvard Medical School, Boston, MA, USA; d W. M. Keck Structural Biology Laboratory, Cold Spring Harbor Laboratory, Cold Spring Harbor, NY, USA

**Keywords:** SARS-CoV-2, antibody, neutralization, severe, non-severe

## Abstract

We studied plasma antibody responses of 35 patients about 1 month after SARS-CoV-2 infection. Titers of antibodies binding to the viral nucleocapsid and spike proteins were significantly higher in patients with severe disease. Likewise, mean antibody neutralization titers against SARS-CoV-2 pseudovirus and live virus were higher in the sicker patients, by ∼5-fold and ∼7-fold, respectively. These findings have important implications for those pursuing plasma therapy, isolation of neutralizing monoclonal antibodies, and determinants of immunity.

The coronavirus disease 2019 (COVID-19) is an infection caused by a newly discovered coronavirus, severe acute respiratory syndrome coronavirus 2 (SARS-CoV-2). Together with SARS-CoV, which caused an outbreak 17 years ago, SARS-CoV-2 is a member of the subgenus *Sarbecovirus*. Both viruses express a glycoprotein termed spike protein (S), which mediates viral entry into ACE2-positive host cells and is therefore the target of virus-neutralizing antibodies [1]. Another structural protein is the nucleocapsid protein (NP), which is the most abundant and highly immunogenic protein in coronaviruses, making it a suitable candidate for diagnostic assays [2].

A study of antibody responses to SARS-CoV-2 in patients with COVID-19 showed that nearly all patients developed virus-specific antibodies within 2–3 weeks after symptom onset [3]. Most serologic studies [3–5] largely focused on binding antibodies to S and NP, but not virus-neutralizing antibodies even though such antibodies can be used therapeutically or prophylactically. Infusion of convalescent plasma has been used to treat SARS-CoV-2 [6]. The measurement of neutralizing antibodies is critical in finding the best donors for plasma therapy, as well as being the gold standard to evaluate vaccine responses. Recent vaccine and re-infection studies in non-human primates suggest that neutralizing antibodies are the correlate of protection against SARS-CoV-2 [7,8]. Studies using convalescent plasma to treat SARS-CoV-2 infections were performed only on a limited number of patients, and there were no careful measurements of neutralizing antibody titers to correlate with the clinical outcome [9]. This study examines SARS-CoV-2 neutralizing antibodies in the plasma of patients with different disease severity.

We studied 35 patients seen at Columbia University Irving Medical Center with PCR-confirmed SARS-CoV-2 infection to assess their plasma antibody responses to the virus. The age, sex, and time of blood collection after onset of symptoms for each patient are summarized in Supplementary Table 1. Patients who required hospitalization in the intensive care unit (19) were categorized as Severe, whereas those with milder disease with or without hospitalization (16) were categorized as Non-severe. As expected, Severe cases were older (range 34–84; mean 58) than Non-severe cases (range 20–58; mean 38). Importantly, blood collection was taken, on average, about one month after the onset of symptoms in both groups.

Immunoassays to quantify antibodies to SARS-CoV-2 NP and S trimer were used to measure binding antibody titers in plasma of both Severe and Non-severe patients. Plasma titers of antibodies to SARS-CoV-2 NP and S trimer were substantially higher in Severe patients than in Non-severe patients (Figure 1a). Specifically, for NP-directed antibodies, the reciprocal plasma titers ranged from 292 to 37,099 (mean 5086) for Severe cases and from 170 to 1376 (mean 615) for Non-severe cases (Figure 1b). The mean plasma titer was ∼8-fold higher in the Severe group, and this difference was statistically significant (*p* = 0.036). Similarly, for S trimer-directed antibodies, the reciprocal plasma titers ranged from 257 to 18,397 (mean 2985) for Severe patients and from <100 to 1963 (mean 364) for Non-severe patients (Figure 1b and Supplementary Table 1). The mean plasma titer was also ∼8-fold higher in the Severe group, and this difference was again statistically significant (*p* = 0.016).

**Figure 1.**
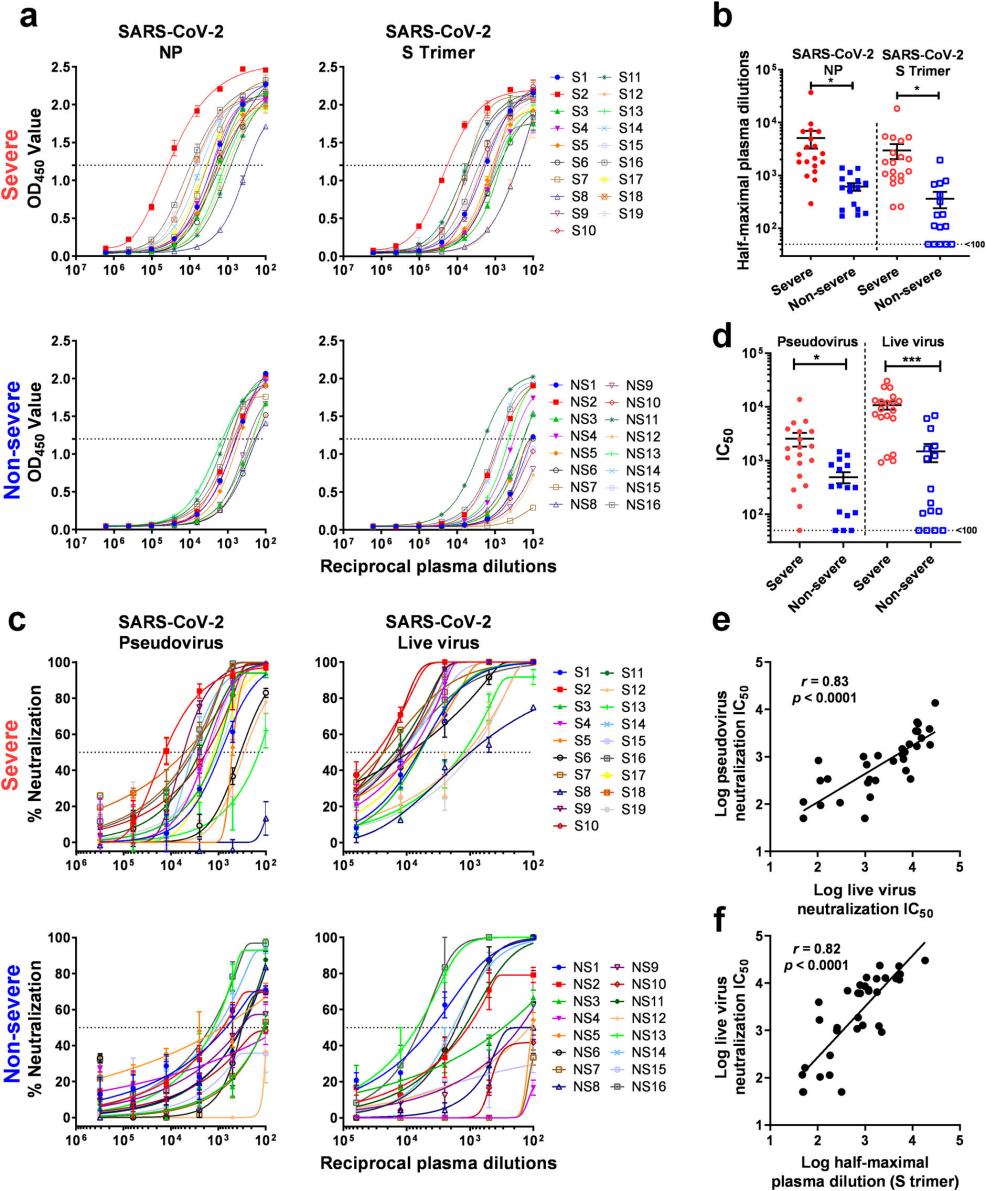
Antibody responses against SARS-CoV-2. (*a*) Plasma titers of binding antibodies to SARS-CoV-2 NP and S trimer in Severe and Non-severe patients. (*b*) Comparison of the level of binding antibodies against SARS-CoV-2 between Severe and Non-severe patients. (*c*) Plasma neutralizing activities against SARS-CoV-2 pseudovirus and live virus in Severe and Non-severe patients. (*d*) Comparison of the level of neutralizing antibodies against SARS-CoV-2 between Severe and Non-severe patients. (*e–f*) Correlation of SARS-CoV-2 live virus neutralization titers versus pseudovirus neutralization titers (*e*) and S trimer-binding antibody titers (*f*). Lines in (*b*) and (*d*) represent mean ± SEM and *p* values were calculated by two-tailed *t*-test. * *p* < 0.05; *** *p* < 0.001. In (*e–f*), the *Pearson correlation coefficient (r)* and the probability *p* value were calculated using GraphPad Prism.

Next, antibody neutralization assays against SARS-CoV-2 pseudovirus and live virus (2019-nCoV/USA_WA1/2020) were performed on plasma samples from Severe and Non-severe patients. Overall, both SARS-CoV-2 pseudovirus and live virus neutralization titers were substantially higher in the plasma of Severe patients compared to those of Non-severe patients (Figure 1c). Specifically, in the SARS-CoV-2 pseudovirus assay, the reciprocal plasma neutralizing titers ranged from <100 to 13,710 (mean 2545) for Severe cases and from <100 to 1463 (mean 491) for Non-severe cases (Figure 1d and Supplementary Table 1). The mean pseudovirus neutralizing titer was ∼5-fold higher in the Severe group, and this difference was statistically significant (*p* = 0.015). Similarly, in the SARS-CoV-2 live virus assay, the reciprocal plasma titers ranged from 926 to 30,175 (mean 10,701) for Severe patients and from <100 to 6884 (mean 1485) for Non-severe patients (Figure 1d and Supplementary Table 1). The mean live virus neutralizing titer was ∼7-fold higher in the Severe group, and this difference was again statistically significant (*p* < 0.001).

A few other findings were notable. First, the plasma neutralizing titers against the SARS-CoV-2 pseudovirus correlated quite well with the titers obtained against the live virus (Figure 1e). In addition, neutralizing titers correlated well with S trimer-binding antibody titers as determined by quantitative immunoassay (Figure 1f).

The results of this study show that patients with severe SARS-CoV-2 disease have more robust binding antibodies to both NP and S trimer (Figure 1a,b). Functionally active antibodies capable of virus neutralization were also more abundant in the sicker patients (Figure 1c,d). The latter finding is reminiscent of the observation that HIV-1 broadly neutralizing antibodies were most commonly found in patients with persistent viremia for a protracted period [10]. There is evidence that patients with severe SARS-CoV-2 infection have a higher viral load [5], and perhaps a longer exposure to a greater abundance of viral antigens is the basis for our findings. Regardless, the results reported herein do have important implications for donor selection when pursuing plasma therapy or isolating neutralizing monoclonal antibodies. Of course, this selection is best made by assessing virus-neutralizing activity in the serum or plasma of potential donors. However, even an S trimer-based immunoassay could provide useful guidance in choosing convalescent patients who have the most robust neutralizing antibodies (Figure 1f). The scientific community and general public eagerly await data that could answer whether having virus-neutralizing antibodies is equivalent to having protective immunity. The strong correlations observed here between antibodies that bind the S trimer and antibodies that neutralize the virus could facilitate future studies to understand what constitutes immunity against SARS-CoV-2.

## Supplementary Material

Clean_copy_of_supplementary_files.docx
